# Ultrasonographic characterization and prognostic follow-up of fetal cardiac involvement in maternal immune diseases

**DOI:** 10.3389/fmed.2025.1564873

**Published:** 2025-06-16

**Authors:** Shaoli Yin, Qi Lin, Shaoting Huang, Jinfeng Xu, Yujuan Zhang

**Affiliations:** Department of Ultrasound, Shenzhen People’s Hospital (The Second Clinical Medical College, Jinan University, The First Affiliated Hospital, Southern University of Science and Technology), Shenzhen, China

**Keywords:** fetus, ultrasound, maternal immune diseases, arrhythmia, endocardial fibroelastosis

## Abstract

**Objective:**

To analyze the clinical characteristics, echocardiographic features, and prognosis of fetuses with maternal immune diseases.

**Methods:**

We retrospectively evaluated 20 fetuses with cardiac manifestations due to maternal immune diseases from our center between 2019 and 2023 in China. Clinical and echocardiographic data of fetuses and maternal sero-immunity were collected. The pregnancy outcomes were followed up.

**Results:**

The cardiac manifestations seen in 20 fetuses were categorized into three types: type I: isolated-arrhythmia: seven cases (35%); type II: isolated endocardial fibroelastosis (EFE): four cases (20%); and type III: both arrhythmia and EFE: nine cases (45%). The arrhythmias in all cases were bradyarrhythmia, including sinus bradycardia and atrioventricular block. The results of maternal antibody test showed the following three types: (1) five cases were positive for anti-SSA antibody alone; (2) 10 cases of positive for both anti-SSA and anti-SSB antibody; (3) five cases of other special types of antibodies. Ultimately, four newborns were delivered and 16 fetuses were terminated. Of the mothers who chose to induce labor, three were treated and repregnant, two of which had normal fetuses, while one had a recurrence of arrhythmia, which was treated and monitored closely during the prenatal period, resulting in normalization of the fetal heart rhythm and eventual full-term delivery.

**Conclusion:**

Fetal cardiac involvement due to maternal immune diseases can be categorized into three types: the isolated-arrhythmia, isolated EFE, and both arrhythmia and EFE. In our study, all cases of arrhythmia were bradyarrhythmias, with sinus bradycardia being the most prevalent type. Given the strong association between maternal autoimmune diseases and fetal cardiac abnormalities such as bradyarrhythmias and EFE, prenatal ultrasound findings of these conditions should raise suspicion for maternal immune disorders. Consequently, prompt screening for maternal autoimmune antibodies is recommended.

## Introduction

Clinically, more than half of autoimmune antibody-positive pregnant women are asymptomatic, and pregnant women with autoimmune diseases are often not diagnosed timely since immune antibody testing has not yet become a routine prenatal screening program (1). However, the antibodies can pass through the placenta and then cause damage to the fetus. Complications include cytopenia, skin rashes, liver damage and even neuropsychiatric disorders, the most important of which is cardiac damage, including autoimmune congenital heart block (ACHB), endocardial fibroelastic hyperplasia (EFE), dilated cardiomyopathy (DCM), and valvular disease (1–5), which can jeopardize the life of the fetus in severe cases. In this study, we summarized the ultrasound signs of cases of fetal cardiac involvement in maternal immune diseases first diagnosed in Shenzhen People’s Hospital in recent years, and followed up the clinical interventions and prognosis, in order to provide a basis for early diagnosis and intervention in pregnant women with autoimmune diseases and perinatal management of their second pregnancy.

## Subjects and methods

### Subjects

From 2019 to 2023, we retrospectively collected 20 fetuses with cardiac manifestations due to maternal immune diseases. The study was approved by the Ethics Committee of Shenzhen People’s Hospital. All pregnant women signed written informed consent prior to their inclusion into this study.

Inclusion criteria: complete case history, positive indicators of maternal immune disorders combined with fetal heart involvement.

Exclusion criteria: incomplete case data, negative indicators of maternal immune disorders, fetal cardiac anomalies unrelated to maternal immune disorders.

### Methods

#### Fetal echocardiography

Two- and three-dimensional ultrasound systems Voluson E8 and Voluson E10 with 4–8 MHz transabdominal curved array probes were adopted in the study. Fetal bradycardia was defined by a heart rate less than 110 bpm. The prolongation of the A-V interval (>150 ms) was considered first-degree AVB. Second-degree AVB showed the AV block of one or more, but not all. Type 1 second-degree AVB manifested a progressive prolongation of the A-V interval until one A wave failed to conduct to the ventricle. Intermittent non-conducted A waves without prolongation of the A-V interval were defined as type 2 second-degree AVB. The absence of A-V conduction was defined as third-degree AVB (6). EFE was defined as bright and white locations of endocardial echogenicity, with clearly defined margins. Sites of mild EFE mainly enriched the surface of the atrial wall, atrioventricular valve, and atrial septum or semilunar valve. Sites of severe EFE should include the surface of the left or right ventricles (7). DCM was identified as left or right ventricular enlargement with a shortening fraction <25% (8).

#### Data collection and follow-up

Clinical data, including family history, maternal antibody profiles (including anti-SSA, anti-SSB, etc.), antenatal therapy, pregnancy outcome and postnatal therapy were collected. After cardiac manifestations were found, the pregnant women were referred to the Rheumatology Unit for standard treatment and regular echocardiographic examinations. Live-born infants were followed up until 12 months after birth.

#### Pathologic examination methods

For terminated fetuses, pathologic examination was performed by a pathologist. Fetal heart specimens were fixed in 4% neutral formaldehyde after autopsy, and paraffin sections were prepared routinely. HE staining was performed, and the histological changes of cardiomyocytes were observed by light microscopy (×100).

## Results

### General condition of fetus and mother

The mean maternal age was 32.25 ± 4.07 years (range 25–40 years). The average gestational age at diagnosis with cardiac manifestations was 26.25 ± 4.75 weeks (range 18–36 weeks). The mean gestational age when a fetal arrhythmia was diagnosed was 25.56 ± 4.76 weeks in the study (range18–32 weeks). All individuals who opted to continue their pregnancies received pharmacological treatment, which included hydroxychloroquine and immunoglobulin. Among the mothers who opted to induce labor, three cases experienced systematic management following labor induction.

### Characteristics of fetal cardiac involvement

**Figure 1:**
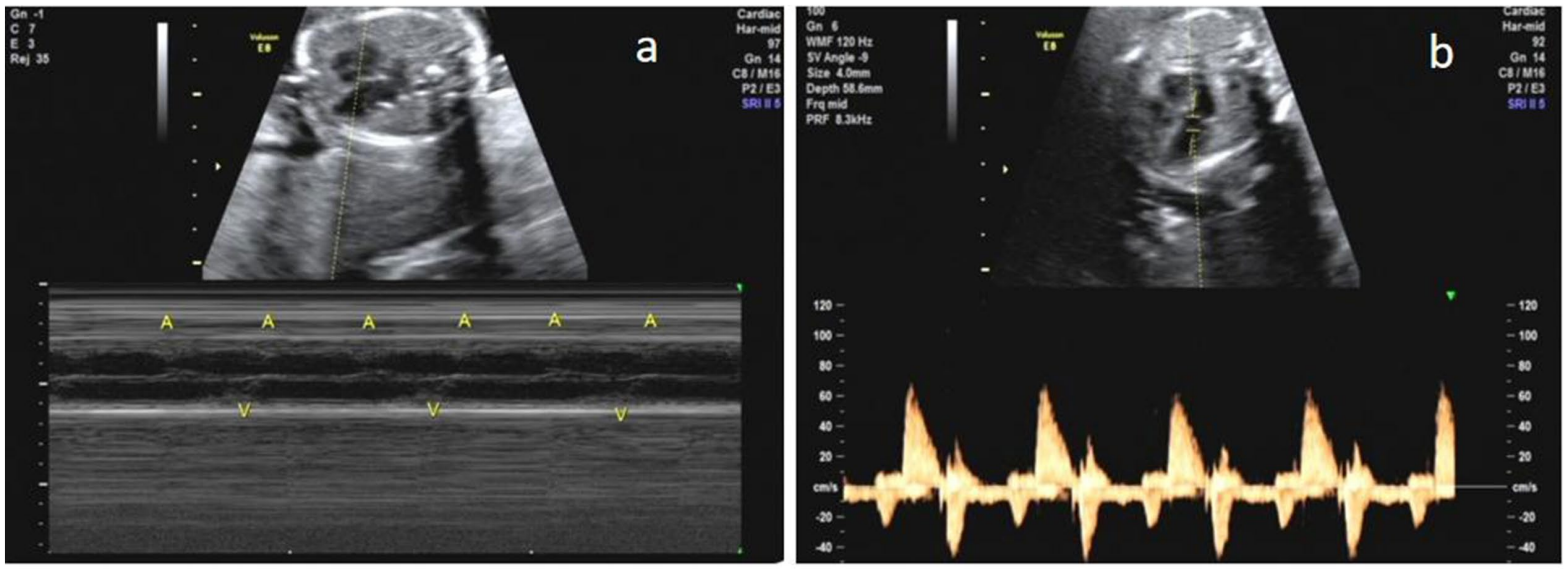
Degree II atrioventricular block. **(a)** M-type. **(b)** PW.

**Figure 2:**
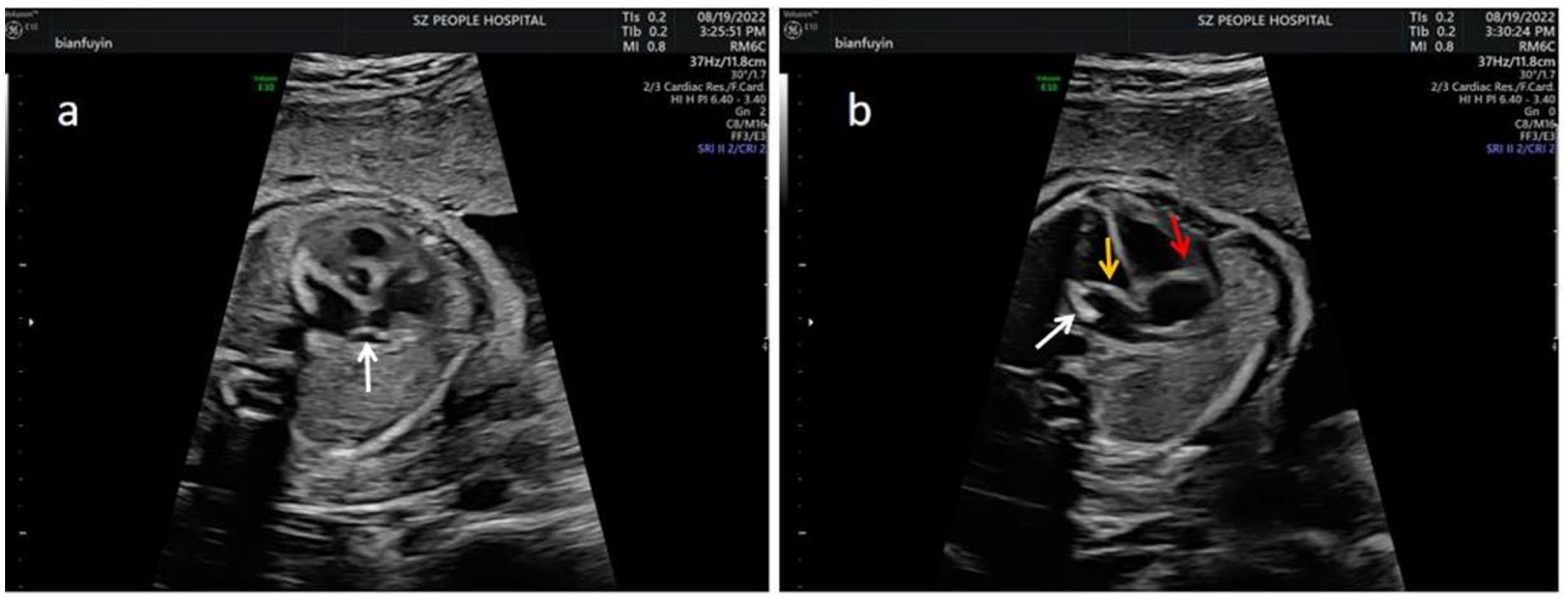
The echocardiographic features of isolated mild EFE in different site. **(a)** Left atrial appendage (white arrow). **(b)** MV (yellow arrow), TV (red arrow) and left atrial side wall (white arrow).

**Figure 3:**
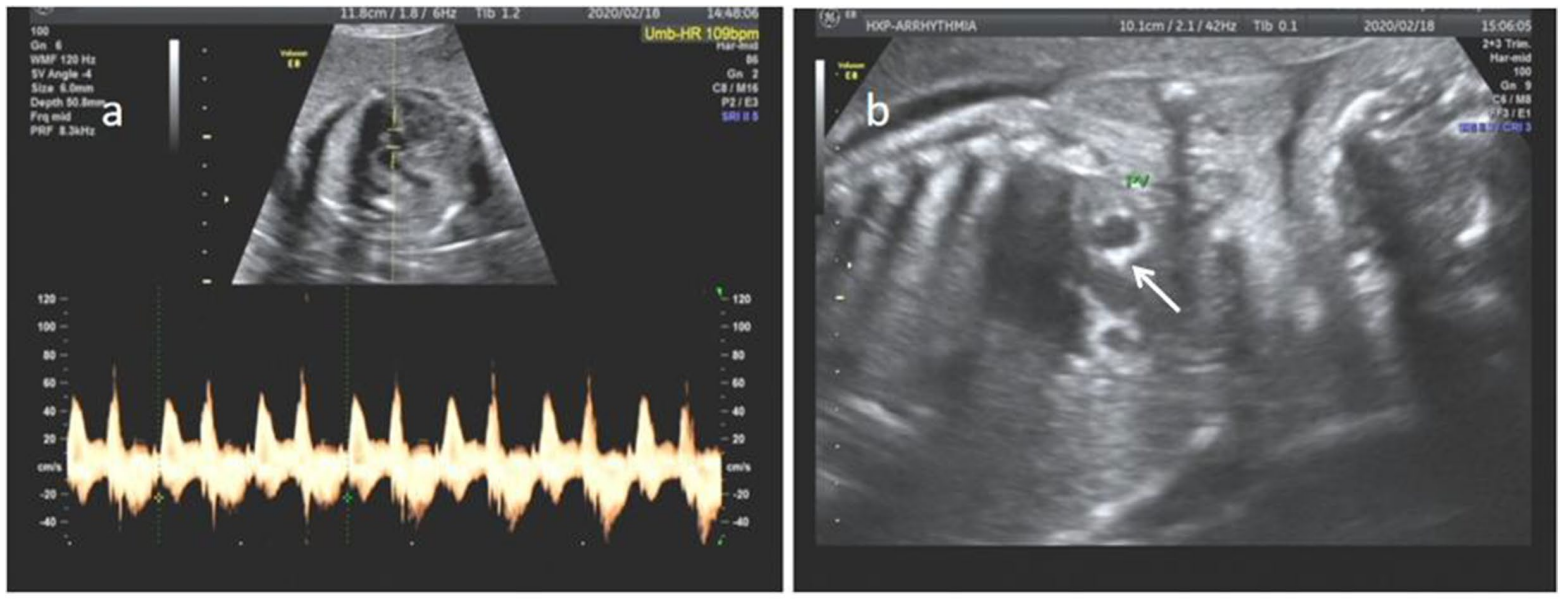
**(a)** Sinus bradycardia, heart rate 109 bpm. **(b)** Pulmonary valve echo enhancement (white arrow).

Arrhythmias were detected in 80% of fetuses (16/20), EFE in 65% (13/20). The cardiac manifestations seen in 20 fetuses were categorized into three types (Table 1): type I: isolated-arrhythmia (Figure 1): seven cases (35%); type II: isolated EFE (Figure 2): four cases (20%); and type III: both arrhythmia and EFE (Figure 3): nine cases (45%). One of the three fetuses who were the second birth showed type I, the other two fetuses were normal.

**Table 1:** Category of fetal cardiac involvement.

Fetal cardiac involvement	Number
Isolated-arrhythmia (type I)	7
Isolated EFE (type II)	4
Both arrhythmia and EFE (type III)	9

#### Arrhythmia characteristics

The arrhythmias in all cases were bradyarrhythmias, including sinus bradycardia and atrioventricular block. Of the 16 identified arrhythmias, there were seven cases of sinus bradycardia, one case of first-degree atrioventricular block, four cases of second-degree atrioventricular block and four cases of third-degree atrioventricular block (Table 2).

**Table 2:** Types of arrhythmia and the number of cases.

Arrhythmia	Type I	Type III	Total
Sinus bradycardia	3	4	7
First-degree AVB	0	1	1
Second-degree AVB	2	2	4
Third-degree AVB	2	2	4

Type I is isolated arrhythmia, and type III include both arrhythmia and EFE.

#### Characteristics of EFE

A total of 13 fetuses exhibited EFE in the study. EFE was observed across an extensive array of anatomical sites, encompassing the mitral valve, tricuspid valve, pulmonary valve, aortic valve, atrial septum, left and right atrial walls, left atrium, and coronary sinus (as detailed in Table 3). The most frequently affected sites included the mitral valve, tricuspid valve, pulmonary valve, atrial septum, and the walls of the left and right atria. The degree of EFE varied from slight enhancement in certain instances to significant enhancement in one particular case, accompanied by localized eggshell changes. All patients in type II presented with mild EFE. Among the nine patients in type III, eight exhibited mild EFE, while one presented with severe EFE.

**Table 3:** Involved sites and number of cases of EFE.

Site	Number of cases
Tricuspid valve	9
Mitral valve	8
Pulmonary valve	5
Atrial septum	4
Left atrial wall	2
Right atrial wall	2
Ventricular septum	1
Aortic valve	1
Left atrial appendage	1
Coronary venous sinus	1

#### Other combined abnormalities

##### Two-dimensional ultrasound

Ten cases exhibited pericardial and peritoneal effusions, including four cases classified as type I, one case as type II, and five cases as type III. Among these, nine fetuses presented with a small amount of pericardial effusion; of these, two also had a small amount of peritoneal effusion, while another fetus exhibited a small to moderate amount of pericardial effusion. Additionally, six fetuses demonstrated an enlarged heart, and one fetus showed incomplete myocardial densification.

##### Color Doppler examination

A total of 13 fetuses exhibited valve regurgitation, comprising four cases of type I, three cases of type II, and six cases of type III. Among these, five cases presented with moderate tricuspid regurgitation, two cases with mild tricuspid regurgitation, and six cases with a small amount of tricuspid regurgitation. Additionally, one case demonstrated moderate mitral regurgitation, while another case exhibited a small amount of mitral regurgitation.

##### Spectral Doppler examination

One case of type III showed venous catheter α-wave reversal.

### Maternal antibody test results

The results of maternal antibody testing revealed three distinct types: five cases were positive for anti-SSA antibodies alone, 10 cases were positive for a combination of anti-SSA and anti-SSB antibodies, and five cases were positive for other specific types of antibodies. These specific types of antibodies included anticardiolipin antibody positivity in three cases and dsDNA antibody positivity in two cases. The correlation between maternal antibodies and fetal cardiac involvement was presented in Table 4. Notably, the three mothers who gave birth to second children tested positive for both anti-SSA and anti-SSB antibodies. Importantly, none of the pregnant women underwent relevant examinations or targeted treatments prior to pregnancy, as they exhibited no obvious clinical symptoms. Maternal immune diseases identified included: systemic lupus erythematosus (SLE) in three cases, Sjögren’s syndrome in six cases, rheumatoid disease in one case, and an unspecified type in 10 cases.

**Table 4:** Correlation between maternal antibodies and fetal cardiac involvement.

Maternal antibody	Type I	Type II	Type III	Total
Anti-SSA(+) alone	1	1	3	5
Anti-SSA(+) anti-SSB(+)	3	3	4	10
Anticardiolipin antibody(+)	3			3
dsDNA antibody(+)			2	2

### Pregnancy outcomes and follow-up results

In this study, 16 fetuses were terminated, while four fetuses were delivered at full term. Among the delivered fetuses, there were three cases of type II and one case of type I identified prenatally. Three of these fetuses exhibited normal prenatal heart rhythms, and in two cases, the echogenic enhancement of the annulus was no longer evident on cardiac ultrasound. In one case, however, the echogenicity of the annulus, the left atrial wall, and the interatrial septum remained enhanced on cardiac ultrasound at 12 months post-birth. One fetus was born with third-degree atrioventricular block and is currently awaiting elective pacemaker implantation. Among the mothers who opted to induce labor, three cases were treated and subsequently became pregnant again. Of these, two cases resulted in normal fetuses, while one case experienced arrhythmia once more. Following treatment and close dynamic monitoring during the prenatal period, the fetal cardiac rhythm normalized, and the fetus was ultimately delivered at full term. Two individuals with fetal arrhythmia ultimately underwent cesarean delivery. Three individuals, driven by personal anxiety, requested cesarean sections. The remaining three cases successfully proceeded with vaginal deliveries.

### Pathological findings

In one case (1/16), the abnormal fetus was autopsied after termination of pregnancy, and there were no obvious abnormalities in the gross view. However, histologic examination showed large calcifications in the myocardium of the interatrial septum.

## Discussion

Clinically, the diagnosis of maternal immune disorders is often delayed. In most cases, fetal cardiac abnormalities are detected prenatally before a definitive diagnosis is established. Maternal immune disorders can cause a variety of damages to the fetus, with the heart being the most prominently affected organ. Autoimmune-mediated damage to the fetal heart is most commonly manifested as ACHB, which is predominantly associated with maternal anti-SSA/Ro antibodies and may be accompanied by elevated anti-SSB(La) antibodies. Maternal Ro/La autoantibodies traverse the placenta and damage the conduction tissue of the fetal heart, resulting in inflammation, fibrosis, and calcification of the atrioventricular (AV) node, thereby causing conduction block (1). The pathogenesis of immune-mediated fetal EFE remains unclear. Many scholars believe that ACHB promotes EFE due to prolonged bradycardia. In recent years, it has been suggested that EFE and ACHB are two independent yet related manifestations of immune-mediated heart disease (3). Maternal autoantibody deposition in the myocardium may also contribute to the development of EFE.

### Analysis of the types and characteristics of fetal arrhythmias

In this study, a total of 16 cases of arrhythmias were identified, comprising seven cases of sinus bradycardia, one case of first-degree AVB, four cases of second-degree AVB, and four cases of third-degree AVB. Among these, nine cases were associated with EFE, while the remaining seven cases were isolated arrhythmias. Previous literature has reported that third-degree AV block is the most common type ACHB (9–14). However, in our study, sinus bradycardia was the most frequently observed arrhythmia, which is inconsistent with prior reports. This discrepancy may be attributed to the limited sample size of our study. Future research should aim to increase the sample size to validate these findings more reliably.

Recent studies have shown that sinus bradycardia is a definite and persistent manifestation of maternal anti-SSA and anti-SSB antibody involvement in the fetal heart, either purely or in combination with AVB (2). In this study, sinus bradycardia was the most common fetal arrhythmia. Previous reports have suggested that calcification of the atrial wall and septum may involve the sinus node and conduction system in corresponding areas, leading to poor or absent sinus node function (15, 16). However, in the present study, three cases of sinus bradycardia were identified without concomitant enhancement of atrial wall, septal, and annular echogenicity. This finding raises questions about the applicability of the aforementioned conclusions in this context.

It is important to note that prenatal ultrasound assessment of fetal arrhythmias is typically achieved using M-mode or spectral Doppler ultrasound to demonstrate atrial and ventricular mechanical activity (16). However, these methods do not evaluate whether the beats in the atrial wall are generated by sinus node conduction. Therefore, sinus bradycardia identified prenatally may manifest as a junctional or atrial rhythm after birth (2, 17). This phenomenon underscores the importance of continued postnatal monitoring and evaluation to accurately characterize the underlying rhythm abnormalities.

### Ultrasound characterization of EFE

Prenatal ultrasound signs of EFE typically include endocardial thickening and echo enhancement, as well as lesions involving the endocardium, atrial wall, and annulus. The severity of EFE can be classified as mild, moderate, or severe based on the thickness and extent of the affected areas (2, 17). Previous studies (3, 18, 19) have shown that fetuses with EFE associated with positive maternal anti-SSA and anti-SSB antibodies tend to present with severe EFE. This severe form is characterized by ventricular endocardial thickening and echo enhancement, often accompanied by ventricular enlargement and reduced cardiac function. Additionally, these cases frequently co-occur with complete AVB. Conversely, the findings of the present study did not align with those reported in the preceding literature. In this study, only one EFE fetus presented with ultrasound signs of ventricular intima-media thickening, echogenic enhancement, increased myocardial gaps, and bradycardia, yet cardiac function was preserved. The other fetuses with extensive and multiple cardiac echogenic enhancements exhibited atypical features compared to severe EFE. The primary sites of involvement included the valve annulus, atrial septum, and atrial wall, while the ventricular chamber internal diameters and functions were normal. These results are somewhat inconsistent with those documented in previous studies (8, 20). Currently, some scholars (7, 21) have reported similar cases as observed in the present study, identifying several instances of echogenic enhancement of the left atrial wall and atrioventricular valve annulus of the fetal heart due to positive maternal anti-SSA and anti-SSB antibodies. These alterations were categorized as a mild-moderate degree of EFE. However, the criteria for this classification of EFE severity are not clearly defined and have not yet gained widespread acceptance. Further studies are needed to establish more precise diagnostic criteria and to better understand the clinical implications of these findings.

### Summary of prenatal ultrasound features of fetal cardiac involvement in maternal immune disease

In this study, three types of fetal cardiac anomalies were identified: type I was isolated arrhythmia; type II was isolated EFE; and type III was the coexistence of arrhythmia and EFE. Notably, type III was the most prevalent in our study, which is consistent with the findings of previous research (8). This observation aligns with the understanding that EFE often presents with other cardiac anomalies, such as arrhythmias, highlighting the complexity and variability of these conditions. Initially, some scholars (22, 23) reported cases of EFE combined with ACHB and suggested that the possible pathogenesis was ACHB due to prolonged bradycardia promoting EFE. In 2002, Nield et al. (3) reported 13 cases of children with complete ACHB and concomitant EFE, primarily involving the left ventricle. All of these cases had severe ventricular dysfunction, leading to death in nine cases and heart transplantation in two cases. In the same year, three cases of severe EFE with positive maternal anti-SSA/Ro antibodies but without ACHB were also reported. Thus, the scholars proposed that EFE and ACHB are two separate but related manifestations of immune-mediated heart disease. The results of the present study support this conclusion. However, the sample sizes of the current studies are limited, and further large sample studies are needed.

### Effects and mechanisms of maternal anti-SSA and anti-SSB antibodies on fetal heart

Maternal anti-SSA and anti-SSB antibodies can pass through the placenta during pregnancy and affect the fetal heart, resulting in structural or rhythmic abnormalities, such as atrioventricular block, EFE, sinus bradycardia, and delayed dilated cardiomyopathy (4). Fetuses with complete AVB and/or EFE have a poor prognosis (2, 24). Positivity of maternal anti-SSA antibody is more likely to result in fetal cardiac EFE and AVB. In addition to being associated with antibody-mediated inflammatory response and myocardial injury, it may be related to its ability to affect calcium channel activity, leading to intracellular calcium deposition in cardiomyocytes, impaired cardiomyocyte function, and apoptosis (17, 25). In the present study, the highest percentage of anti-SSA antibody positivity was 15/20, suggesting a more pronounced association between fetal cardiac involvement and maternal anti-SSA antibodies. This finding is consistent with the results reported in the literature (17, 25).

However, five cases exhibited positivity for specific maternal antibodies: three cases were positive for anticardiolipin antibodies, and two cases were positive for dsDNA antibodies. Meanwhile, all five cases were negative for anti-SSA and anti-SSB antibodies. Cardiac involvement of the five case comprised three cases of type III and two cases of type I. To date, few research has investigated the effects of dsDNA and cardiolipin antibodies on fetal cardiac. Our study suggests that these two antibodies may potentially contribute to fetal heart involvement. However, given the small sample size, additional studies are warranted to validate these findings.

### Prognosis

It has been reported in the literature that fetuses with normal heart rhythms but widespread echogenic enhancement in the atrial wall and annulus have a favorable short-term prognosis following birth, with ultrasound signs of echogenic enhancement potentially resolving by 4 to 7 years of age (17). However, whether late-onset DCM may occur in the long term remains to be determined through large-scale, long-term studies. Fetuses with first-degree AVB can either revert to a normal heart rhythm or persist with first-degree AVB after birth (25). In contrast, fetuses with complete AVB are at a higher risk of intrauterine demise and typically require pacemaker implantation postnatally (7).

In this study, the overall prognosis was not very good, with 16 fetuses being induced. Only four cases were delivered at term: three had normal prenatal heart rhythms, and two showed resolution of annular echogenic enhancement on cardiac ultrasound by 12 months postpartum. One case continued to exhibit increased echogenicity in the annulus, left atrial wall, and atrial septum. Another fetus was born with third-degree AVB, stabilized with treatment, and is scheduled for elective pacemaker installation. What’s more, among the mothers who opted to induce labor, three cases were treated and subsequently became pregnant again. Of these, two cases resulted in normal fetuses, while one case experienced arrhythmia once more. Fortunately, following treatment and close dynamic monitoring during the prenatal period, the fetal cardiac rhythm normalized, and the fetus was ultimately delivered at full term. The current condition of these fetuses is stable, but their long-term prognosis remains uncertain and requires further follow-up. Brito-Zerón et al. (1) emphasized the importance of maternal screening for autoimmune antibodies when fetal or neonatal echogenic foci or severe valvular regurgitation are detected. Our study concluded that prenatal ultrasound findings of no significant ventricular endocardial abnormalities but multiple echogenic enhancements in the atrial wall, septum, and annulus, or arrhythmias, also warrant testing for maternal autoimmune antibodies.

### Limitations

The main limitations of our study include its small size and the absence of histological confirmation in most of the induced cases.

In summary, characteristics of fetal cardiac involvement in maternal immune diseases can be classified into three types: isolated arrhythmia, isolated EFE, both arrhythmia and EFE. All the arrhythmias cases were bradyarrhythmias, and sinus bradycadia was the largest type. Most EFE cases were mild. Combined with the results of maternal antibody profiling, early detection and timely intervention in prenatal period is of certain significance in minimizing the incidence of adverse pregnancy outcomes.

## Data Availability

The raw data supporting the conclusions of this article will be made available by the authors, without undue reservation.
